# Detection of immunoglobulin G levels produced by oral polio vaccine in HIV infected children in Jos, Plateau State, Nigeria

**DOI:** 10.11604/pamj.2019.34.183.16665

**Published:** 2019-12-06

**Authors:** Fwangshak Ayuba Lengkat, Onuoha Stanley Chukwudozie, Oladele Olasoji Vincent, Dasun Martin James, Hashimu Godiya Amina, Onyedibe Kenneth

**Affiliations:** 1 Department of Medical Microbiology, Faculty of Medical Sciences, University of Jos, P.M.B 2084 Jos, Plateau State, Nigeria; 2 Department of Biotechnology, Ebonyi State University, Abakaliki, Ebonyi State, Nigeria; 3 Department of Educational Services, Universal Basic Education, P.M.B 163 Garki, Abuja, Nigeria

**Keywords:** Immunoglobulin G, oral polio vaccine, human immunodeficiency virus

## Abstract

**Introduction:**

Disease eradication requires a long time and efficient management as compared to disease control program. After successful small pox eradication, polio virus causing poliomyelitis is choice for next eradication. The corner stone of the global polio eradication initiative is the immunization of children with multiple doses of Oral Polio Vaccine (OPV) through both Routine Immunization (RI) and Supplemental Immunization Activities (SIAs). This informed our design of this prospective study. Objective is to determine levels of Immunoglobulin G antibodies produced in HIV infected children aged (one to ten years) vaccinated with Oral Polio Vaccine (OPV) in Jos, Plateau State, Nigeria.

**Methods:**

One hundred and eighty-two children infected with HIV who had received Oral Polio Vaccine (OPV) at least four times had their blood samples collected and assayed for the presence of Polio Specific IgG antibodies using IgG ELISA test kit (DEMEDITEC Diagnostic GmbH, Germany). Three millilitre (3ml) of venous blood samples were collected aseptically by venepuncture. Sera obtained were assayed using Enzyme immunoassay detection and quantitative determination of human IgG antibodies against poliomyelitis virus in serum and plasma (Demeditic Poliomyelitis Virus IgG ELISA DEPOL01-Germany).

**Results:**

The result showed that 95.6% (174/182) of the tested children had detectable IgG antibodies against polio virus. The high proportion of 95.6% recorded in this study indicates HIV infected children responded effectively to the Oral Polio Vaccine (OPV) being used in the ongoing polio eradication initiative. In this study, 4.4% (8/182) of the HIV infected children were not producing detectable amount of antibodies that could protect them from exposure to wild type of polio virus.

**Conclusion:**

This study shows that HIV infected children had detectable antibodies (Immunoglobulin G) against polio virus. Despite the overall progress recorded in the fight against poliomyelitis in Nigeria, a lot needs to be done to further strengthen the fight against poliomyelitis in Nigeria.

## Introduction

After successful small pox eradication, polio virus causing poliomyelitis became the next focus for eradication [1,2]. Poliomyelitis is a highly infectious disease that mainly affects children under five years of age. It invades the nervous system and can cause total paralysis in a matter of hours. The wild types are of three (3) known serotypes (1, 2, and 3). The virus is transmitted from person to person and spread mainly through the faecal-oral route or less frequently by a common vehicle (for example contaminated water and food) and multiplies in the intestine [3]. For symptomatic cases, initial symptoms are fever, fatigue, headache, vomiting, stiffness of the neck and pain in the limbs. One in every 200 infections leads to irreversible paralysis. In the presence of paralysis, death occurs in 5 to 10% when their breathing muscle become immobilized. There is no cure for poliomyelitis. It can only be prevented. Polio vaccine given multiple times can protect a child for life [4,5]. In May 1988, the 41st World Health Assembly adopted a resolution to globally eradicate polio by the year 2000 which led to the launching of the Global Polio Eradication Initiative (GPEI), a public-private partnership led by the World Health Organization (WHO), Rotary International, the U.S Centres for Disease Control and Prevention (CDC), and the United Nation International Children Emergency Fund (UNICEF). The Bill and Melinda Gates foundation have also supported this initiative generously with massive funding [6]. With this initiative and concerted effort, through the use of immunization with polio vaccine, the world has witnessed a remarkable reduction in paralytic poliomyelitis cases from 350,000 in more than 125 countries in 1988 to 247 cases in 10 countries as at October 2014 [7]. The worldwide sustained use of polio vaccine since 1988 has led to a reduction in the number of cases of poliomyelitis by more than 99% globally.

The corner stone of the global polio eradication initiative is the immunization of children with multiple doses of Oral Polio Virus Vaccine (OPV) [7,8]. The key advantage in the usage of OPV was the ease of administration and the efficient reduction of mucosal colonisation thereby limiting polio shedding and person to person transmission of polio virus [9]. The risk of an adverse event after receiving Oral Polio Vaccine (OPV) by HIV infected children is low, but there have been cases of children with primary immunodeficiency syndromes (such as B-cell disorders or X-linked agammaglobulinemia) who developed vaccine associated paralytic polio (VAPP) after receiving OPV. Inactivated Polio Vaccine (IPV) is considered the safer choice and is used for HIV infected children and household contact in countries where it is available. The WHO continues to recommend the use of OPV in infants and children with unknown HIV status or for HIV infected children who are asymptomatic in resource limited areas. Symptomatic HIV infected children can receive IPV. There are no immediate side effects directly linked to OPV administration. Vaccine associated paralytic polio usually occurs within 2 months after immunization, but the risk is low and estimated to be at a rate of 1:7.8 million IPV doses [10]. In order to assess the effectiveness of OPV in conferring protective immunity against polio virus infection in HIV infected children, we quantitatively determined the Immunoglobulin G (IgG) antibody levels produced in HIV infected children vaccinated with Oral Polio Vaccine (OPV) in Jos, Plateau State, Nigeria.

## Methods

**Study location:** the study location was Jos which is the capital of Plateau State. Plateau State is one of the 36 States of Nigeria, covering a land area of 11.936km^2^. It has 17 Local Government Areas with a population of 3.179 million people. The State has three geo-political zones namely; Plateau North, Plateau Central, Plateau South. This study was carried out in Plateau North. These two hospitals; Faith Alive Foundation and Aids Preventive Initiative in Nigeria APIN/JUTH are selected for the study because they carry out health care services for HIV/AIDS patients. They are President Emergency Plan for AIDS Relief (PEPFAR) clinics in Jos.

**Study design:** this is a cross sectional study; a study in which a group of individuals are selected on the basis of factors that are to be examined for possible outcome.

**Study population and duration:** all children between the ages of one to ten years infected with HIV and have been vaccinated with Oral Polio Vaccine. The study lasted between August 2016 to March 2018.

**Inclusion criteria:** HIV infected children within the ages of one to ten years attending Jos University Teaching Hospital (JUTH)-APIN, and Faith Alive Foundation in Jos, Plateau State whose parents or guardian gave their informed consent.

**Exclusion criteria:** HIV infected children whose age did not fall within the age group 1-10 years. Children whose parent or guardians refuse to give consent for their enrolment in the study. Children of HIV negative mothers and children of HIV positive mothers with other debilitating illnesses such as malignancy, diabetes mellitus and sepsis.

**Ethical clearance:** ethical clearance was obtained from Jos University Teaching Hospital (JUTH) and Faith Alive Foundation, ethical clearance committees before embarking on the exercise. Consents obtained from parent/guardian after explaining what the study is all about before enrolling the children for the study.

**Sample size:** the sample size of the study was determine using the formula:

$$N=\frac{Z^{2}\left(PQ\right)}{D^{2}}$$

at 95% confidence level. A report of prevalence by (97.8%) of IgG antibodies in children aged 1 to 7 in Jos. Where N= sample size, Z= statistics for a level of 95% confidence level= 0.95, P= prevalence rate of IgG from previous study= 97.8%= 0.978 (Dashe *et al*. [11]). D= level of significance= 7.5%= 0.075, Q= 1-P, substituting in the formula: N= (0.95)2*0.978(1-0.978)/0.072= 0.9025*0.978*0.9022/0.005625= 0.796322319/0.005625= 141.569 from the above formula the minimum sample size is 142, 10% attrition= 14.16+142= 155. However, a total of 182 samples were examined.

**Sample collection:** a questionnaire was given to the parent/guardian of each child to retrieve relevant information. After parental consent to participate in the study, about 3ml of blood sample was aseptically drawn from each child by venepuncture. The specimen was collected into a labelled sterile container free of anticoagulant or preservatives allowed for clotting. Subsequently, samples were transported to the laboratory immediately in a cold box with ice packs to maintain cold chain at about 4-80c. Serum was recovered from each sample by low speed centrifugation at 500g for 5 minutes followed by direct removal of the serum using a sterile disposable pipette. The serum was transferred into labelled sterile cryo vials per sample and stored at -800c until ready for analysis (DEMEDITEC Diagnostic GmbH Germany).

**Sample processing/laboratory analysis:** the analysis was carried out at Plateau State Institute of Human Virology (PLASVIREC). The polio IgG antibody ELISA test kit manufactured for the detection of specific IgG antibodies against polio in serum of children with strict adherence to manufacture manual was used. (DEMEDITEC Diagnostic GmbH Germany).

**Data/statistical analysis:** the data obtained in this study were analysed using Statistical Package for Social Science (SPSS) computer software program. Pearson Chi-square was used to test for association between discrete variables. Statistical significance was accepted at P<0.05 (95%) confidence level.

## Results

A total of 182 children aged 1-10 years' male and female infected with HIV had their blood samples collected and analysed for the presence of polio specific IgG antibodies using IgG ELISA test kit (DEMEDITEC Diagnostic GmbH, Germany) (Table 1). The proportion of HIV infected children aged 1 to 10 years who developed IgG antibodies to polio virus following vaccination from the two clinics tested showed that APIN/JUTH had positive prevalence of 96.7%, while FAITH ALIVE clinic had 94.5%. The overall percentage prevalence of polio specific IgG antibody among the study population was found to be 95.6% (Table 2). The result of the distribution of Polio IgG antibodies concentration among HIV infected children shows that concentration <10 u/ml were negative, while those ≥10 u/ml were positive (Table 3). The result for concentration 10-19 u/ml was 83(45.6%), 20-29 u/ml was 78(42.9%) and concentration <30u/ml was 13(7.1%), all were positive (Table 3). This difference in the sero-positivity could be due to the number of children sampled and analysed with concentration <30 having the lowest seropositivity and concentration 10-19 u/ml having the highest seropositivity.

**Table 1:** Doses of Oral Polio Vaccines (OPV) received

Vaccine Doses	Number of Children Tested
At Birth (OPV_0_)	182
6 Weeks (OPV_1_)	182
10 Weeks (OPV_2_)	182
14 Weeks (OPV_3_)	182

**Table 2:** Proportion of HIV infected children aged 1 to 10 years who developed IgG antibodies to polio virus following vaccination

	APIN/	FAITH	Total	Overall
IgG Ab	JUTH (%)	ALIVE (%)	Frequency	Percentage (%)
Positive	88(96.7)	86(94.5)	174	95.6
Negative	3(3.3)	5(5.5)	8	4.4
**Total**	91	91	182	100.0

t= 0.38, df= 180, P-value=0.71; * P > 0.05 – not statistically significant

**Table 3:** Distribution of polio IgG antibodies concentration among children aged 1-10 years infected with HIV in Jos Plateau State

IgG Ab Conc. (u/ml)	Frequency	Percentage
0-9	8	4.4
10-19	83	45.6
20-29	78	42.9
>30	13	7.1
Total	182	100

KEY: (0-9.99) u/ml = Negative; ≥10u/ml = Positive

The result of gender as a risk factor was also determined in this study as shown in Table 4. The highest prevalence of 96.7% (87/90) was observed among female children as compared to 94.6% (87/92) recorded among the male counterpart. But, there was no statistically significant association between sex and seropositivity to the polio virus. Age as a risk factor for polio virus infection was also determined in this study (Table 5). The higher seropositivity of 95.9% (140/146) was found among older children aged 6-10 years as compared to seropositivity of 94.4% (34/36) found among younger children aged 1-5 years (Table 5). The result of the association between polio IgG and level of father's education: χ^2^ = 1.95; df = 3; p-value = 0.58: thus P > 0.05 and association between polio IgG and level of mother's education: χ^2^ = 1.67; df = 3; p-value = 0.64: thus, P > 0.05 were not statistically significant as shown in Table 6. The result from Table 7, shows the association between Polio IgG and sources of drinking water and association between Polio IgG and type of toilet facility.

**Table 4:** Distribution of polio IgG antibodies among children aged 1-10years infected with HIV in relation to gender

Sex	No. tested	No. positive IgG	Percentage positive IgG
Male	92	87	94.6
Female	90	87	96.7

n= 182, t = 0.48, df = 1, P-value =0.49; *P>0.05 not statistically significant; male to female ratio: 1:1

**Table 5:** Age distribution of IgG antibodies among children aged 1-10 years infected with HIV

Age (yrs) Percentage	No tested	No. positive IgG	positive IgG
1 – 5	36	34	94.4
6 – 10	146	140	95.9

t= 0.38, df= 180, P-value=0.71; * P > 0.05 – not statistically significant

**Table 6:** Distribution of polio IgG antibodies among children aged 1-10 years infected with HIV in relation to parents occupation

Father	Mother	n=182
Occupation Of parents	IgG Positive Frequency (%)	IgG Positive Frequency (%)
Civil servant	65 (48.9)	44 (25.6)
Artisan	23 (17.3)	7 (4.1)
Trader	64 (48.1)	66 (38.4)
Farmer	9 (6.8)	5 (2.9)
Other	11 (8.3)	4 (2.3)
Unemployed	1 (0.8)	
Housewife		46 (26.7)

Association between polio IgG antibodies and father’s occupation: χ2 = 4.46; df = 1; p-value = 0.62; thus **P > 0.05** – not statistically significant.

Association between polio IgG antibodies and mother’s occupation: χ2 = 2.59; df = 6; p-value = 0.86; thus **P > 0.05** – not statistically significant.

**Table 7:** Distribution of polio IgG antibodies among children aged 1-10 years infected with HIV in relation to source of drinking water and type of toilet facility

Factor	Positive IgG Frequency	n=182 (Percentage)
**Source of drinking water**		
Pipe borne	64	(36.8)
Private well	43	(24.7)
Public well	4	(2.3)
Borehole	49	(28.2)
Others	14	(8.0)
**Total**	174	**(100)**
**Type of toilet facility**		
Pit	56 (32.2)	
Water system	102 (58.6)	
Field	13 (7.5)	
Others	3 (1.7)	
**Total**	**174 (100)**	

Association between Polio IgG and sources of drinking water: **P > 0.05** – not statistically significant. (**χ2= 1.06, df= 4, P-value= 0.90);** Association between Polio IgG and type of toilet facility: **P > 0.05** – not statistically significant. (**χ2= 2.75, df= 3, P-value= 0.43)**

## Discussion

From the results obtained from the proportion of HIV infected children who developed IgG antibodies to polio virus following vaccination. The high prevalence of 95.6% recorded in this study indicates children responded effectively to the OPV being used in the polio eradication initiative (Table 2). This finding was higher than the 73.6% prevalence by [12]. However, a higher prevalence of 97.8% was obtained in Jos [11]. The difference in the seroconversion might be due to: immune suppression as in the case of this study, low proportion of full vaccination in the country, concurrent enteroviral infection, interference among serotypes of OPV and poor maintenance in cold chain and sub-optimal practice of vaccine handling. The result of polio IgG antibodies concentration shows that concentration <10 u/ml were negative, while those ≥10 u/ml were positive. The negative result as obtained from the proportion of HIV infected children who developed IgG antibodies to polio virus following vaccination implies that 8 of the children are not producing Immunoglobulin G despite being immunized. This might be because the children were having some form of immune impairment like HIV as in the subject of this study or the mothers must have given a wrong information of the child being vaccinated. The children that are not producing immunoglobulin G are at risk of re-infection and continued transmission of the virus in the community. The result of gender as a risk factor was also determined in this study as shown in Table 4. The result showed that gender had no effect on the overall prevalence of polio virus antibody. This is to say that both male and female children had equal chances of being immunized during routine immunization sessions and also equal chances of exposure to natural infection. This agrees well with what had been observed by [13].

The result of age as a risk factor shows that the higher seropositivity was found among older children as compared to seropositivity found among younger children aged 1-5 years (Table 5). The result could be deduced to more doses of the oral polio vaccine and to declining exposure to circulating oral polio vaccine. Also, age naturally determines the number of doses taken either through routine immunization or during campaigns. This finding is in agreement with [14] who stated that increase in immunity level has earlier been associated with age. The result of the association between polio IgG and level of father's and mother's education were not statistically significant. The result is dissimilar to the work of [12]. This might be because of intense campaign through immunization plus days where children are followed in homes, schools, churches, and play grounds with the oral polio vaccine. As a result, parents whose children never had any form of formal education had detectable antibodies to polio virus. This reflects that fact that irrespective of educational status, the general population has been sensitized on the immunization of children and may be attributed to the high level of antibody production as showed in this study. The association between polio IgG and sources of drinking water and between polio IgG and type of toilet facility were not statistically significant at P > 0.05. All these are supposedly possible risk factors because polio virus is transmitted from person to person and spread mainly through the fecal oral route or less frequently by a common vehicle (for example contaminated water and food). This further demonstrates the high level of awareness campaign and the effectiveness of the oral polio vaccine in circulation.

## Conclusion

This study shows that HIV infected children had detectable antibodies (Immunoglobulin G) against polio virus. Despite the overall progress recorded in the fight against poliomyelitis in Nigeria, a lot needs to be done in the fight against poliomyelitis.

### What is known about this topic

- All studied population had appreciable levels of protection against polio virus due to levels of antibodies detected;
- No significant association between detection of IgG in children in relation to gender and age;
- Educational status of parent had statistically significant relationship with detection of antibodies in children.

### What this study adds

- Despite the fact that the vaccine works with the immune system of an individual, HIV infected children (immune compromised children) were able to produce detectable level of antibodies (immunoglobulin G) against polio virus;
- This research work demonstrated the progress that has been made towards the eradication of poliomyelitis in Nigeria.

## Competing interests

The authors declare no competing interests.
